# Association of *GPx1 P198L* and *CAT C-262T* Genetic Variations With Polycystic Ovary Syndrome in Chinese Women

**DOI:** 10.3389/fendo.2019.00771

**Published:** 2019-11-08

**Authors:** Yuan Sun, Suiyan Li, Hongwei Liu, Yan Gong, Huai Bai, Wei Huang, Qingqing Liu, Linbo Guan, Ping Fan

**Affiliations:** ^1^Laboratory of Genetic Disease and Perinatal Medicine, Key Laboratory of Birth Defects and Related Diseases of Women and Children, Ministry of Education, West China Second University Hospital, Sichuan University, Chengdu, China; ^2^School of Life Science and Engineering, Southwest Jiaotong University, Chengdu, China; ^3^Department of Obstetrics and Gynecology, West China Second University Hospital, Sichuan University, Chengdu, China

**Keywords:** polycystic ovary syndrome, glutathione peroxidase 1, catalase, superoxide dismutase 2, genetic polymorphism, oxidative stress

## Abstract

**Background:** Oxidative stress plays an important role in the pathogenesis of polycystic ovary syndrome (PCOS). Glutathione peroxidase 1 (GPx1*)* and catalase (CAT*)* are the major intracellular antioxidant enzymes that can detoxify hydrogen peroxide into water, preventing cellular injury from reactive oxygen species. The aim of the present study was to investigate the association of *GPx1 P198L* (*Pro198Leu, C559T, rs1050450*) and *CAT C-262T* (*rs1001179*) genetic polymorphisms with the risk of PCOS and evaluate the effects of the genotypes on clinical, hormonal, metabolic and oxidative stress parameters in Chinese women.

**Methods:** This is a case-control study of 654 patients with PCOS and 535 controls. The *GPx1 P198L, CAT C-262T*, and superoxide dismutase 2 (*SOD2*) *A16V* genotypes were determined by polymerase chain reaction amplification and restriction analysis. Clinical, hormonal, metabolic and oxidative stress parameters were also analyzed.

**Results:** The frequencies of the *PL* + *LL* genotype (14.1 vs. 8.4%) and *L* allele (7.3 vs. 4.4%) of *GPx1 P198L* polymorphism were significantly higher in patients with PCOS than in control subjects. Genotype (*PL* + *LL*) remained a significant predictor for PCOS in prognostic models including age, body mass index (BMI), insulin resistance index, total cholesterol, triglycerides, high-density lipoprotein-cholesterol, and low-density lipoprotein-cholesterol as covariates (OR = 2.105, 95%CI: 1.330–3.331, *P* = 0.001). Patients carrying the *L* allele had relatively high average ovarian volume, waist circumference, and malondialdehyde levels (*P* < 0.07) compared with patients with the *PP* genotype. We also demonstrated that the subjects with both *GPx1 L* and *SOD2 A* alleles further increase the risk of PCOS compared with the individuals carrying the *PP/VV* genotype after adjusting for age and BMI (OR = 5.774, 95%CI: 2.243–14.863, *P* < 0.001). However, no significant differences were observed in the frequencies of the *CAT C-262T* genotypes and alleles between PCOS and control groups.

**Conclusions:** The *GPx1 P198L*, but not *CAT C-262T*, genetic polymorphism is associated with the risk of PCOS in Chinese women.

## Introduction

Polycystic ovary syndrome (PCOS) is a heterogeneous female endocrine-reproductive-metabolic abnormality with a worldwide prevalence of 4–21% in reproductive-aged women, depending on diagnostic criteria (1, 2). In addition to reproductive disorders, PCOS is often associated with overweight, obesity and central obesity, dyslipidemia, insulin resistance (3–5), increased oxidative stress, chronic low-grade inflammation (6–8), elevated risks of metabolic syndrome, impaired glucose tolerance, type 2 diabetes, and future cardiovascular diseases (3, 9, 10). The etiology of PCOS is unclear, but studies have suggested that PCOS appears to have a complex, multifactorial etiology resulting from the interactions between genetic, environmental, and intrauterine factors (3).

Glutathione peroxidase (GPx) 1, the most common isoform of the GPx family, is a selenium-dependent antioxidant enzyme that can reduce free hydrogen peroxide (H_2_O_2_) to water by using glutathione (GSH) as a reducing substrate in human cells. Thus, GPx1 plays an important role in the detoxification of H_2_O_2_ and a wide range of organic peroxides (11, 12). *GPx1 P198L (Pro198Leu, C559T, rs1050450)* polymorphism is a *C* to *T* substitution in exon 2 of *GPx1* gene, leading to an amino acid change from proline (Pro) to leucine (Leu) at codon 198 (12), which results in a reduced enzyme activity (13). *GPx1* gene *P198L* polymorphism has been reported to be related with some oxidative stress-related diseases, such as lung cancer (12), breast cancer (13), bladder cancer (14), diabetic peripheral neuropathy (11), and inflammatory bowel disease (15).

Catalase (CAT) is another antioxidant enzyme that detoxifies H_2_O_2_ into oxygen and water, preventing cellular injury from reactive oxygen species (16). Hereditary catalase deficiencies have been associated with increased risk of diabetes (17). A common functional single-nucleotide polymorphism (SNP) *C-262T (rs1001179)* in the promoter region of the human *CAT* gene influences transcription factor binding, resulting in a lower *CAT* gene expression in the *C* allele compared to the *T* allele (18). However, the association between the *C-262T* polymorphism and CAT activity is contradictory, but most report a decrease in activity associated with the *T* allele (19, 20). The *C-262T* polymorphism has been reported to be related to some human diseases, such as prostate cancer (21), male infertility (22), and glucose and lipid abnormalities in patients with type 2 diabetes or hyperlipidemia (19, 20).

Oxidative stress plays a key role in the occurrence and development of PCOS (8), so understanding the function of SNPs involved with antioxidant enzymes is fundamental. Our previous studies have demonstrated that 3 SNPs of the antioxidant enzymes, the *Q192R* variant in the paraoxonase 1 (*PON1*) gene, the *G994T* polymorphism in the platelet-activating factor acetylhydrolase (*PAF-AH*) gene, and the *A16V* variant in the superoxide dismutase 2 (*SOD2*) gene are associated with the risk of PCOS in Chinese women (23–25). Because GPx1 and CAT are important intracellular antioxidant enzymes and play an important role in maintaining the dynamic balance of oxidative stress interacting with SOD2 (26, 27), we hypothesized that the functional SNPs, the *P198L* in *GPx1* gene and the *C-262T* in *CAT* gene, may be associated with PCOS. Salahshoor et al. (28) reported that the *GPx1 P198L* and the *CAT C-262T* SNPs were not associated with the risk of PCOS in the Kurdish population, but it remains unknown whether there are any relationships between these 2 gene polymorphisms and PCOS in Chinese women. In the present study, we investigated the relationships between *GPx1 P198L* and *CAT C-262T* variants and the risk of PCOS, evaluated the effects of the genotypes on clinical, hormonal, metabolic and oxidative stress parameters, and analyzed the associations of the genotype combinations of *GPx1 P198L* and *SOD2 A16V* with PCOS in well-characterized Chinese women using relatively large sample sizes.

## Subjects and Methods

### Study Subjects

This was a case–control study with 654 cases and 535 controls. Women with or without PCOS aged 17–40 years were recruited from 2006–2019 from the Outpatient Clinic of Reproductive Endocrinology of the West China Second University Hospital. All of the participants provided their written informed consent, and the study was approved by the Institutional Review Board of the West China Second University Hospital, Sichuan University (2014-014 to PF).

Each patient with PCOS met the revised 2003 Rotterdam ESHRE/ASRM consensus criteria (29). Oligo-ovulation or anovulation (OA) was assessed as irregular menstrual cycles (i.e., <21 or >35 days or <8 cycles per year) (30). Clinical or biochemical hyperandrogenism (HA) was assessed by hirsutism with a modified Ferriman–Gallwey (F–G) score of more than 6, clinical presence of obvious acne, total testosterone (TT) levels and/or free androgen index (FAI) above the 95th percentile of the levels (TT ≥ 2.60 nmol/L, FAI ≥ 9.5) detected in a group of normal menstruating women with normal cycles (4, 23, 31). Polycystic ovaries (PCOs) were confirmed if there were 12 or more follicles in each ovary measuring 2–9 mm in diameter and/or increased ovarian volume (>10 mL) by ultrasonic examination. The diagnosis of PCOS was based on a patient having 2 of these 3 findings, with HA as an essential condition for women aged <20 years (30, 32) and exclusion of other etiologies, such as androgen-secreting tumors, congenital adrenal hyperplasias, and Cushing syndrome. All the controls had regular menstrual cycles (between 21 and 35 days), exhibited normal circulating androgen levels, did not show hirsutism or obvious acne on physical examination, and had normal ovarian morphology as determined by ultrasound.

None of the subjects had clinically evident acute or chronic diseases, such as infection, tumors, cardiovascular disease, thyroid dysfunction, autoimmune diseases, endometriosis, premature ovarian insufficiency, hyperprolactinemia, or hypogonadotropic hypogonadism.

The subjects were also excluded if they had one of the following interferential factors: (i) taking hormones or medication (such as oral contraceptives, metformin, etc) known to affect the metabolism of carbohydrates or lipids within 3 months before the study; (ii) being pregnant or in the luteal phase because progesterone was reported to influence oxidative stress (33, 34); and (iii) smoking.

Clinical and anthropometric variables, including waist circumference, hip circumference, waist-to-hip ratio, body mass index (BMI, kg/m^2^), systolic and diastolic blood pressure (SBP and DBP), and the degree of acne and hirsutism were measured or evaluated as described previously (35, 36) in all subjects. Ultrasound ovarian volume (ml) on each side was calculated using the formula for the volume of an ellipsoid (37): 0.523 × length (cm) × width (cm) × thickness (cm).

Blood samples were obtained in the morning after overnight fasting, placed on ice immediately and centrifuged at 1,500 g for 15 min at 4°C within 2 h. Plasma or serum were stored at −80°C, and blood cells were stored at 4°C.

### Genotype Analysis

Genomic DNA was extracted from whole blood samples as previously described (24, 25). The *GPx1 P198L* and *CAT C-262T* genotypes were determined by polymerase chain reaction (PCR) amplification and restriction analysis according to the method of Kasznicki et al. (38) with some modification. A total volume of 25 μl containing 2.5 μl of 10X PCR buffer, 200 μM each of dNTP, 1.5 mM MgCl_2_, and 0.75 U of Taq polymerase (Thermo Fisher Scientific Inc. Vilnius, Lithuania), 1.5 μl of genomic DNA template (about 30–80 ng), 0.30 μM of each primer: forward 5′-GTGCCCCTACGCAGGTACAG-3′ and reverse 5′-GGACATACACACAGTTCTGCTGAC-3′ for the *P198L* genotype (designed by Primer-BLAST); forward 5′-CTGATAACCGGGAGCCCCGCCCTGGGTTCGGATAT-3′ and reverse 5′-CTAGGCAGGCCAAGATTGGAAGCCCAATGG-3′ for the *C-262T* genotype were used (39). The PCR was performed as follows: pre-denaturation at 95°C for 5 min, followed by 33 (GPx1)/29 (CAT) cycles of 45 s at 95°C, 45 s at 66°C (GPx1)/70°C (CAT), and 45 s at 72°C, and ending with a single 7 min extension step at 72°C. Five microliter of the *P198L* (319 bp) or *C-262T* (190 bp) PCR products were digested with 8U of ApaI or 5U of EcoRV (New England Biolabs, Inc.) in 10 μl of reaction volume for 1–3 h at 25 or 37°C, respectively, and digestion resulted in 239- and 80-bp fragments for the *198P* allele and in a non-digested 319-bp fragment for the *198L* allele, 157- and 33-bp fragments for the *-262C* allele, and a non-digested 190-bp fragment for the *-262T* allele. The products were analyzed by electrophoresis on a 2.5 or 3.5% agarose gel and visualized by staining with Genecolour fluorescent dye. *SOD2 A16V* polymorphisms (*C47T, rs4880*) were measured as described previously (23). For the purpose of genotyping quality control, more than 30% of DNA samples were randomly genotyped again by a different operator and the results of the two genotyping were identical.

### Analysis of Hormonal, Metabolic, and Oxidative Stress Parameters

Serum follicle-stimulating hormone (FSH), luteinizing hormone (LH), TT, sex hormone-binding globulin (SHBG), estradiol (E_2_), total cholesterol (TC), triglyceride (TG), HDL-cholesterol (HDL-C), LDL-cholesterol (LDL-C), total oxidant status (TOS), total antioxidant capacity (T-AOC) and malondialdehyde (MDA) levels, plasma insulin and glucose concentrations, as well as free androgen index (FAI), homeostatic model assessment of insulin resistance (HOMA index), and oxidative stress index (OSI) were measured or assessed as described previously (4, 6, 23). The intra- and inter-assay coefficients of variation for all measurements were <5 and 10%, respectively.

### Statistical Analyses

Data were presented as the mean ± standard deviation (SD). Differences in variables were evaluated by the independent sample *t*-test between PCOS and control subjects. Variables with asymmetric distribution were evaluated by non-parametric tests (Mann–Whitney *U*-test). Chi-squared (*x*^2^) analysis was used to test deviations of genotype distribution from Hardy–Weinberg and to determine allele or genotype frequencies between patients and controls. Odds ratios (ORs) and 95% confidence intervals (CIs) were calculated to test relative risk for PCOS associated with the gene variants by *x*^2^ analysis and logistic regression methods. Analysis of covariance was used to estimate the differences in metabolic and oxidative stress parameters between 2 groups after correction for differences in covariates, such as age and BMI. A *P*-value <0.05 was considered to be statistically significant. All statistical analyses were performed using the Statistical Program for Social Sciences (SPSS) 13.0 for Windows (Chicago, IL, USA).

## Results

### Clinical and Biochemical Characteristics of the Study Population

As shown in Table 1, BMI was significantly increased and age was significantly decreased in the PCOS group compared with the control group.

**Table 1:** Clinical, hormonal, metabolic, and oxidative stress parameters in PCOS patients and control women.

	**Controls (*n* = 535)**	**PCOS (*n* = 654)**	***P***	***P^***a***^***
Age (years)	28.15 ± 4.15	25.00 ± 4.21	0.000	
BMI (kg/m^2^)	21.12 ± 2.86	23.24 ± 4.27	0.000	
Waist circumference (cm)	73.67 ± 8.38	80.00 ± 11.42	0.000	0.000
Waist-to-hip ratio	0.81 ± 0.06	0.85 ± 0.07	0.000	0.000
F-G score	0.25 ± 0.73	1.74 ± 2.06	0.000	0.000
Acne grade score	0.14 ± 0.35	0.65 ± 0.91	0.000	0.000
SBP (mmHg)	112.93 ± 11.48	114.84 ± 10.72	0.004	0.976
DBP (mmHg)	73.72 ± 8.86	75.79 ± 8.94	0.000	0.042
AOV (ml)	7.65 ± 2.89	10.11 ± 4.09	0.000	0.000
**Hormonal levels**				
TT (nmol/L)	1.51 ± 0.53	2.35 ± 0.77	0.000	0.000
SHBG (nmol/L)	54.59 ± 27.16	35.12 ± 26.84	0.000	0.000
FAI	3.40 ± 2.11	9.78 ± 7.07	0.000	0.000
LH/FSH	1.25 ± 1.25	2.30 ± 1.28	0.000	0.000
**Metabolic profile**				
Fasting Ins (pmol/L)	63.82 ± 35.26	106.01 ± 72.79	0.000	0.000
Fasting Glu (mmol/L)	5.24 ± 0.47	5.36 ± 0.86	0.002	0.900
HOMA-IR	2.23 ± 1.30	3.81 ± 3.09	0.000	0.000
TG (mmol/L)	1.04 ± 0.88	1.44 ± 1.39	0.000	0.000
TC (mmol/L)	4.25 ± 0.71	4.43 ± 0.82	0.000	0.000
HDL-C (mmol/L)	1.51 ± 0.32	1.38 ± 0.34	0.000	0.002
LDL-C (mmol/L)	2.36 ± 0.63	2.57 ± 0.76	0.000	0.000
**Oxidative stress parameters**				
TOS (nmol H_2_O_2_ Equiv./mL)	11.24 ± 5.32	14.92 ± 10.77	0.000	0.000
T-AOC (U/ml/min)	14.50 ± 2.69	15.80 ± 3.06	0.000	0.000
OSI	0.80 ± 0.41	0.99 ± 0.79	0.000	0.000
MDA (nmol/ml)	3.66 ± 1.07	4.34 ± 1.31	0.000	0.000

*Values are presented as mean ± SD*.

*AOV, average ovarian volume; BMI, body mass index; DBP, diastolic blood pressure; FAI, free androgen index; F-G score, Ferriman–Gallwey score; FSH, follicle-stimulating hormone; Glu, glucose; HDL-C, high-density lipoprotein cholesterol; HOMA index, homeostatic model assessment of insulin resistance; Ins, insulin; LDL-C, low-density lipoprotein cholesterol; LH, luteinizing hormone; MDA, malondialdehyde; OSI, oxidative stress index; SBP, systolic blood pressure; SHBG, sex hormone-binding globulin; T-AOC, total antioxidant capacity; TC, total cholesterol; TG, triglycerides; TOS, total oxidant status; TT, total testosterone*.

*P^a^, All comparisons of parameters were corrected for differences in age and BMI between the 2 groups except the parameters of age and BMI*.

Waist circumference, waist-to-hip ratio, F-G score, acne grade score, DBP, average ovarian volume, TT levels, the ratio of LH to FSH, FAI, fasting insulin concentration, HOMA index, TG, TC, LDL-C, TOS, T-AOC, and MDA levels, and OSI were significantly higher, whereas SHBG and HDL-C levels were significantly lower in the PCOS group compared with the control group after adjusting for age and BMI (Table 1).

### Distribution of *GPx1 P198L* and *CAT C-262T* Genotypes and Alleles

Genotypic and allelic frequencies of *GPx1 P198L* and *CAT C-262T* polymorphisms are summarized in Table 2. Genotypic distributions were in Hardy–Weinberg equilibrium in patients with PCOS and the control women (all *P* > 0.05). The frequencies of the *PL* + *LL* genotype (14.1 vs. 8.4%) and *L* allele (7.3 vs. 4.4%) of *GPx1 P198L* polymorphism were significantly higher in the PCOS group compared with the control group (OR = 1.671, 95% CI: 1.190–2.346, *P* = 0.003). Logistic regression analysis adjusted for age, BMI, HOMA-IR, TG, TC, HDL-C and LDL-C showed that the genotype (*PL* + *LL*) remained a significant predictor for PCOS (OR = 2.105, 95%CI: 1.330–3.331, *P* = 0.001). No significant differences were found in the frequencies of *CAT C-262T* genotypes and alleles between PCOS and control groups (Table 2, *P* > 0.05).

**Table 2:** Frequencies of *GPx P198L (Pro198Leu)* and *CAT C-262T* genotype and allele in PCOS patients compared with control women.

		**Controls**	**PCOS**	***x^2^***	***P***
		**(*n* = 535)**	**(*n* = 654)**		
**Genotype**
*GPx 198*	*PP*	490 (91.6%)	562 (85.9%)		
	*PL*	43 (8.0%)	88 (13.5%)		
	*LL*	2 (0.4%)	4 (0.6%)	9.235	0.010
*CAT−262*	*CC*	499 (93.3%)	614 (93.9%)		
	*CT*	35 (6.5%)	38 (5.8%)		
	*TT*	1 (0.2%)	2 (0.3%)	0.433	0.805
**Allele frequency**
*GPx 198*	*P*	0.956	0.927		
	*L*	0.044	0.073	9.043	0.003^*^
*CAT−262*	*C*	0.965	0.968		
	*T*	0.035	0.032	0.112	0.738

*Data of genotype are presented as number (%) of patients or controls*.

* *Odds ratio (OR) = 1.671, 95% confidence interval (CI): 1.190–2.346*.

### Associations of *GPx1 P198L* and *CAT C-262T, GPx1 P198L* and *SOD2 A16V*, and *CAT C-262T* and *SOD2 A16V* Genotype Combinations With PCOS

Because the sample sizes of the *GPx1 198LL, CAT*−*262TT*, or *SOD2 AA* homozygotes were too small, we combined them into heterozygous subgroups.

As shown in Table 3, for the *GPx1 P198L* and *CAT C-262T* genotype combinations, the frequencies of *the PL*+*LL/CC* combined genotype were 12.8% in patients with PCOS and significantly higher than 7.7% in the control women. The multinomial logistic regression model including age and BMI as covariates showed that the *PL*+*LL/CC* combined genotype is a risk factor for PCOS (OR = 1.745, 95%CI: 1.128–2.701, *P* = 0.012) when the *PP/CC* combined genotypes as the reference category.

**Table 3:** Frequencies of combined genotypes of *GPx1 P198L* and *CAT C-262T, GPx1 P198L* and *SOD2 A16V*, and *CAT C-262T* and *SOD2 A16V* in patients with PCOS and the control women.

**Genotype combinations**	**Controls (*n* = 535)**	**PCOS (*n* = 654)**	**OR**	**95%CI**	***P***
***GPx1 P198L*** **and** CAT C-262T^*^					
*PP/CC*	458 (85.6%)	530 (81.0%)	1.00	–	–
*PL+LL/CC*	41 (7.7%)	84 (12.8%)	1.745	1.128–2.701	0.012
*PP/CT+TT*	32 (6.0%)	32 (4.9%)	0.828	0.468–1.464	0.516
*PL+LL/CT+TT*	4 (0.7%)	8 (1.2%)	1.665	0.438–6.331	0.454
***GPx1 P198L*** **and** SOD2 A16V^**^					
*PP/VV*	384 (71.8%)	417 (63.8%)	1.00	–	–
*PL+LL/VV*	39 (7.3%)	61 (9.3%)	1.333	0.829–2.145	0.236
*PP/AV+AA*	106 (19.8%)	145 (22.2%)	1.300	0.943–1.791	0.109
*PL+LL/AV+AA*	6 (1.1%)	31 (4.7%)	5.774	2.243–14.863	0.000
***CAT C-262T*** **and** SOD2 A16V^***^					
*CC/VV*	393 (73.5%)	452 (69.1%)	1.00	–	–
*CC/AV+AA*	106 (19.8%)	162 (24.8%)	1.421	1.039–1.942	0.028
*CT+TT/VV*	30 (5.6%)	26 (4.0%)	0.745	0.404–1.376	0.347
*CT+TT/AV+AA*	6 (1.1%)	14 (2.1%)	1.880	0.652–5.423	0.243

*Data of genotype combinations are presented as number (%) of patients or controls*.

* *Chi-squared test: x^2^ = 9.558, P = 0.023. Odds ratio (OR) and 95% confidence interval (CI) were calculated in a multinomial logistic regression model including age and BMI as covariates, the PP/CC combined genotypes (wild-type) as the reference category*.

** *Chi-squared test: x^2^ = 17.416, P = 0.001. OR and 95% CI were calculated in a multinomial logistic regression model including age and BMI as covariates, the PP/VV combined genotypes (wild-type) as the reference category*.

*** *Chi-squared test: x^2^ = 7.742, P = 0.058. OR and 95% CI were calculated in a multinomial logistic regression model including age and BMI as covariates, the CC/VV combined genotypes (wild-type) as the reference category*.

For the *GPx1 P198L* and *SOD2 A16V* genotype combinations, the frequencies of the *PL*+*LL/AV*+*AA* combined genotypes were 4.7% in patients with PCOS, and significantly higher than 1.1% in the control women (*P* = 0.001, Table 3). The multinomial logistic regression model including age and BMI as covariates showed that compared with the subjects carrying the *PP/VV* genotype, the individuals carrying both the *GPx1 L (PL*+*LL* genotype*)* and *SOD2 A* (*AV*+*AA* genotype*)* alleles have the highest risk of PCOS (OR = 5.774, 95%CI: 2.243–14.863, *P* < 0.001, Table 3).

For the *CAT C-262T* and *SOD2 A16V* genotype combinations, the frequencies of *the CC/AV*+*AA* combined genotype were 24.8% in patients with PCOS and relatively high than 19.8% in the control women (*P* = 0.058, Table 3). The multinomial logistic regression model including age and BMI as covariates showed that the *CC/AV*+*AA* combined genotype is a risk factor for PCOS (OR = 1.421, 95%CI:1.039–1.942, *P* = 0.028) when the *CC/VV* combined genotypes as the reference category.

### Effects of *GPx1 P198L* and *CAT C-262T* Genetic Variants on Clinical, Hormonal, Metabolic, and Oxidative Stress Parameters

We further analyzed effects of *GPx1 P198L* and *CAT C-262T* genetic variants on oxidative stress as well as clinical, hormonal, and metabolic parameters in PCOS patients and control women.

As shown in Table 4, compared with the *PP* genotype subgroup, the *L* allele carriers (*PL*+*LL* genotype) of the *GPx1 P198L* polymorphism had significantly lower acne grade score (*P* = 0.004) and tended to have increased waist circumference, average ovarian volume, and MDA levels (*P* < 0.07) in the patients with PCOS. The controls with the *L* allele had higher SBP (*P* = 0.025) and tended to have increased OSI (*P* = 0.084) when compared to the controls with the *PP* genotype.

**Table 4:** Clinical characteristics, hormonal levels, and oxidative stress parameters of the *GPx P198L (Pro198Leu)* genotypes in PCOS patients and controls.

	**Controls**		**PCOS**	
	***PP***	***PL+LL***	***PP***	***PL+LL***
	**(*n* = 490)**	**(*n* = 43+2)**	**(*n* = 562)**	**(*n* = 88+4)**
Age (years)	28.12 ± 4.17	28.42 ± 3.97	25.07 ± 4.15	24.60 ± 4.58
BMI (kg/m^2^)	21.14 ± 2.92	20.88 ± 2.23	23.28 ± 4.28	22.94 ± 4.20
Waist circumference (cm)	73.82 ± 8.37	73.29 ± 6.90	79.97 ± 11.45	80.36 ± 11.32
Waist-to-hip ratio	0.81 ± 0.06	0.81 ± 0.06	0.85 ± 0.07	0.85 ± 0.07
F-G score	0.25 ± 0.74	0.29 ± 0.59	1.72 ± 2.04	1.87 ± 2.24
Acne grade score	0.14 ± 0.34	0.16 ± 0.37	0.69 ± 0.92	0.38 ± 0.77^b^
SBP (mmHg)	112.61 ± 11.51	116.50 ± 10.60^a^	114.66 ± 10.81	115.97 ± 10.09
DBP (mmHg)	73.77 ± 8.81	73.14 ± 6.70	75.69 ± 9.08	76.39 ± 8.08
AOV (ml)	7.65 ± 2.99	7.59 ± 1.71	9.96 ± 3.89	10.99 ± 5.03
**Hormonal levels**				
TT (nmol/L)	1.51 ± 0.53	1.58 ± 0.51	2.35 ± 0.78	2.37 ± 0.71
SHBG (nmol/L)	55.14 ± 27.68	46.22 ± 15.33	35.27 ± 28.02	34.25 ± 18.11
FAI	3.27 ± 2.11	3.85 ± 2.22	9.89 ± 7.26	9.10 ± 5.70
LH/FSH	1.24 ± 1.15	1.44 ± 2.00	2.30 ± 1.30	2.27 ± 1.17
**Oxidative stress parameters**				
TOS (nmol H_2_O_2_ Equiv./mL)	11.15 ± 5.18	12.19 ± 6.61	14.82 ± 10.72	15.59 ± 11.12
T-AOC (U/ml/min)	14.52 ± 2.69	14.28 ± 2.78	15.76 ± 3.09	15.99 ± 2.90
OSI	0.79 ± 0.39	0.91 ± 0.57	0.98 ± 0.78	1.02 ± 0.83
MDA (nmol/ml)	3.64 ± 1.06	3.88 ± 1.22	4.31 ± 1.23	4.57 ± 1.71

*Values are presented as mean ± SD*.

*AOV, average ovarian volume; BMI, body mass index; DBP, diastolic blood pressure; FAI, free androgen index; F-G score, Ferriman–Gallwey score; FSH, follicle-stimulating hormone; LH, luteinizing hormone; MDA, malondialdehyde; OSI, oxidative stress index; SBP, systolic blood pressure; SHBG, sex hormone-binding globulin; T-AOC, total antioxidant capacity; TOS, total oxidant status; TT, total testosterone*.

*Comparisons of all parameters were corrected for differences in age and BMI between the 2 subgroups except the parameters of age and BMI*.

a *P < 0.05, compared with the PP genotype subgroup in controls*.

b *P < 0.01, compared with the PP genotype subgroup in PCOS patients*.

In addition, compared with the controls with the *CT* + *TT* genotype, the controls with the *CC* genotype of the *CAT C-262T* polymorphism had higher or relatively high acne grade score and waist circumference (*P* < 0.08) but relatively low F-G score (*P* = 0.065, Table 5). No significant differences were observed in clinical, hormonal, and oxidative stress parameters between the *CC* genotype subgroup and the *CT* + *TT* genotype subgroup in the patients with PCOS (*P* > 0.05, Table 5).

**Table 5:** Clinical characteristics, hormonal levels, and oxidative stress parameters of the *CAT C-262T* genotypes in PCOS patients and controls.

	**Controls**		**PCOS**	
	***CC***	***CT+TT***	***CC***	***CT+TT***
	**(*n* = 499)**	**(*n* = 35+1)**	**(*n* = 614)**	**(*n* = 38+2)**
Age (years)	28.20 ± 4.07	27.44 ± 5.15	25.05 ± 4.24	24.33 ± 3.70
BMI (kg/m^2^)	21.14 ± 2.88	20.84 ± 2.59	23.25 ± 4.26	22.97 ± 4.40
Waist circumference (cm)	73.82 ± 8.37	71.46 ± 8.34	80.10 ± 11.44	78.41 ± 11.28
Waist-to-hip ratio	0.82 ± 0.06	0.80 ± 0.06	0.85 ± 0.07	0.84 ± 0.07
F-G score	0.24 ± 0.67	0.47 ± 1.32	1.73 ± 2.06	1.93 ± 2.14
Acne grade score	0.15 ± 0.37	0.00 ± 0.00^a^	0.64 ± 0.90	0.83 ± 0.96
SBP (mmHg)	113.07 ± 11.48	110.79 ± 11.50	114.82 ± 10.72	115.26 ± 10.69
DBP (mmHg)	73.73 ± 8.71	73.55 ± 8.00	75.68 ± 8.83	77.44 ± 10.41
AOV (ml)	7.58 ± 2.72	8.27 ± 4.39	10.05 ± 3.96	11.13 ± 5.82
**Hormonal levels**				
TT (nmol/L)	1.50 ± 0.53	1.66 ± 0.53	2.35 ± 0.78	2.36 ± 0.74
SHBG (nmol/L)	54.61 ± 27.57	54.37 ± 20.03	34.81 ± 27.07	40.07 ± 22.67
FAI	3.42 ± 2.15	3.14 ± 1.51	9.89 ± 7.08	7.99 ± 6.76
LH/FSH	1.27 ± 1.27	1.10 ± 0.81	2.31 ± 1.30	2.11 ± 0.91
**Oxidative stress parameters**				
TOS (nmol H_2_O_2_ Equiv./mL)	11.17 ± 5.30	12.14 ± 5.49	14.85 ± 10.65	16.16 ± 12.56
T-AOC (U/ml/min)	14.54 ± 2.75	13.97 ± 1.77	15.77 ± 3.09	16.13 ± 2.62
OSI	0.80 ± 0.41	0.88 ± 0.37	0.99 ± 0.80	1.00 ± 0.69
MDA (nmol/ml)	3.66 ± 1.08	3.64 ± 1.02	4.35 ± 1.28	4.21 ± 1.76

*Values are presented as the mean ± SD*.

*AOV, average ovarian volume; BMI, body mass index; DBP, diastolic blood pressure; FAI, free androgen index; F-G score, Ferriman–Gallwey score; FSH, follicle-stimulating hormone; LH, luteinizing hormone; MDA, malondialdehyde; OSI, oxidative stress index; SBP, systolic blood pressure; SHBG, sex hormone-binding globulin; T-AOC, total antioxidant capacity; TOS, total oxidant status; TT, total testosterone*.

*Comparisons of all parameters were corrected for differences in age and BMI between the 2 subgroups except the parameters of age and BMI*.

a *P < 0.05, compared with the CC genotype subgroup in controls*.

No significant differences were observed in metabolic indexes between the *PP* genotype subgroup and the *PL* + *LL* genotype subgroup or between the *CC* genotype subgroup and the *CT* + *TT* genotype subgroup in the patients with PCOS or the control women (*P* > 0.05, data not shown).

## Discussion

In this study, we show that the *GPx1 P198L*, but not the *CAT C-262T*, genetic variant, is associated with a risk of PCOS in Chinese women. We also found that patients carrying the *L* allele carriers (*PL*+*LL* genotype) of the *GPx1 P198L* polymorphism had relatively high average ovarian volume, waist circumference, and MDA levels, but lower acne grade score compared with patients carrying the *PP* genotype, suggesting that increased risk of PCOS in patients with the *P198L* variant may be potentially linked to ovarian functional abnormalities and increasing levels of oxidative stress in patients. Furthermore, we demonstrated that the individuals carrying both *GPx1 198L (PL*+*LL* genotype*)* and *SOD2 16A* (*AV*+*AA* genotype*)* alleles had further increased risk of PCOS compared with the subjects carrying the *PP/VV* genotype after adjusting age and BMI, implying that the combination of the *SOD2 16A* and *GPX1 198L* alleles may have a synergistic effect on the risk of PCOS and patients with PCOS have a genetic susceptibility to oxidative stress.

Oxidative stress plays an important role in the pathogenesis of PCOS (6, 8, 36). The study of SNPs of antioxidant genes is a helpful method to determine the susceptibility of patients to oxidative stress and elucidate the pathogenesis of disease. CAT and GPx1, located in the peroxisomes and mitochondria, respectively, are primarily intracellular antioxidant enzymes, can detoxify H_2_O_2_ into water, and play a key role in protecting cells against the toxic effects of H_2_O_2_ (27, 40, 41). Genetic studies have indicated that the *L* allele of *GPx1 P198L* polymorphism is associated with decreased enzyme activity of GPx1 (13) and is a risk factor for lung cancer among Caucasians (12), breast cancer among Danish women (13), diabetic peripheral neuropathy in the Polish population (11), and coronary artery disease in the Chinese population (42). The *LL* genotype is also associated with increased risk of bladder cancer in the Turkish population (14) and ulcerative colitis (15). The *C-262T* polymorphism in the promoter region of the CAT gene has been reported to affect the expression of *CAT* gene (16, 18, 19) and the *T* allele is associated with lower enzyme activity of CAT although the results reported previously are not entirely consistent (19, 20). Several studies have demonstrated that the CC genotype or *C* allele of *C-262T* polymorphism is a risk factor for diabetic neuropathy in type 1 diabetes in the Russian population (43) and male infertility in the Spanish population (22), while the *T* allele is associated with the risk of increased asthma among Hispanic children (44) and breast cancer (45).

Increased sources of H_2_O_2_ and/or decreased GPxl and/or CAT activities due to genetic variation may result in an increase of H_2_O_2_ both inside and outside cells, relating to the occurrence of some diseases (27, 46, 47). For example, it has been reported that increasing age, smoking, low serum selenium levels, and the *GPx1 P198L* and *CAT C-262T* genetic polymorphisms were significantly associated with prostate disease risk (47). The *GPx1 P198L*, but not *CAT C-262T*, genetic polymorphism was associated with non-Hodgkin's lymphoma (48), while the *CAT C-262T*, but not *GPx1 P198L*, genetic polymorphism was associated with cerebral palsy after perinatal hypoxic-ischaemic encephalopathy (49). In the present study, we found that the prevalence of the *L* allele (*PL* + *LL* genotype) was significantly more frequent in patients with PCOS than in the control women. Genotype (*PL*+ *LL*) remained a significant predictor for PCOS in prognostic models, including age, BMI, HOMA-IR, TG, TC, HDL-C, and LDL-C as covariates. Our results suggest that the *L* allele of *GPx1 P198L* polymorphism is a genetic risk factor for PCOS in Chinese women. However, our study was unable to prove that the *CAT C-262T* genetic polymorphism was associated with the risk of PCOS in Chinese women, which was consistent with the report by Salahshoor et al. (28).

It has been shown that 3 genes are active on the pathway of detoxification of reactive oxygen species (ROS) from O^**·**^_2_^**−**^ to H_2_O_2_ (SOD2), and further to H_2_O (GPX1 and CAT), and the genetic variations of these genes may influence the efficiency of this detoxification (11, 16, 27). Ravn-Haren et al. (13) reported that there is a significant effect of the *GPx1 gene P198L* variant on enzyme activity and the catalytic activity was lowered 5% for each additional copy of the variant *L* allele. Recently, our study found that the *A* allele *SOD2 A16V* polymorphism is a genetic risk factor of PCOS in Chinese women (23). The *SOD2V*→*A* variant has been reported to enhance SOD2 precursor mitochondrial transport and thus increases enzyme activity in the mitochondrial matrix (50), increasing O^**·**^_2_^**−**^ dismutation and decreasing ROS-mediated toxicity in the mitochondria because membrane permeability H_2_O_2_ easily reaches the cytosol (46). However, increased SOD2 activity may lead to cell damage by H_2_O_2_ overproduction, especially in individuals with a decreased capacity to remove this highly toxic ROS by GPx or CAT (46, 51). Based on the association of the *GPx1 198P*→*L* variant with decreased enzyme activity (13) and the increased risk of bladder and breast cancer due to the combination of the *SOD2 Ala/Ala* and *GPX1 Leu/Leu* genotypes (14, 51), we further analyzed association of *SOD2 A16V* and *GPx1 P198L* genotype combinations with the risk of PCOS. The results showed that the individuals carrying the *PL*+*LL/AV*+*AA* combined genotype had further increased risk of PCOS. In addition, we showed that the *PL*+*LL/AV*+*AA* genotype subgroup had higher SBP (118.96 ± 9.22 vs. 114.57 ± 10.25 or 114.78 ± 11.07 mmHg, *P* < 0.05) and DBP (79.29 ± 7.22 vs. 75.03 ± 8.15 or 75.64 ± 9.84 mmHg, *P* < 0.05), higher or relatively high OSI (1.25 ± 1.14 vs. 0.90 ± 0.59 or 0.87 ± 0.54, *P* < 0.08), but lower or relatively low T-AOC levels (14.73 ± 2.64 vs. 16.58 ± 2.85 or 15.77 ± 3.34 U/ml/min, *P* < 0.10) compared with the *PL*+*LL/VV* or *PP/AV*+*AA* genotype subgroups in patients with PCOS. Our results suggest that the combination of the *SOD2 A* and *GPx1 L* alleles may have a synergistic effect on the risk of PCOS.

We should point out that this study has some limitations. Firstly, given the low frequency of homozygosity of minor alleles, *GPx1 198LL*, and *CAT*−*262TT*, we could not analyze them in the form of subgroups. A larger sample size of patients and controls are needed to properly evaluate dose-dependent genotype characteristics. Secondly, we could not determine GPx and CAT activities. Further study to detect these enzyme activities in the patients with different genotypes may help provide clues to the mechanisms responsible for the genetic association.

In summary, we have demonstrated that the *GPx1 P198L*, but not *CAT C-262T* genetic polymorphism is associated with the risk of PCOS in Chinese women. Our findings also suggest that the *P198L* variant increasing the risk of PCOS may be implicated in ovarian functional abnormalities and increased oxidative stress in patients. Furthermore, we indicate that, compared with women carrying both *PP* genotype of *GPx1 P198L* and *VV* genotype of *SOD2 A16V* polymorphisms, the women carrying both the 198*L* allele and *16A* allele had higher risk of PCOS. Our studies suggest that antioxidant enzyme gene variation may increase the sensitivity of patients to oxidative stress and thus contribute to the pathogenesis of PCOS.

## Data Availability Statement

The datasets for this study can be found at the found here: CAT C-262T (rs1001179): https://www.ncbi.nlm.nih.gov/snp/rs1001179, GPx1 P198L (rs1050450): https://www.ncbi.nlm.nih.gov/snp/rs1050450, SOD2 A16V (rs4880): https://www.ncbi.nlm.nih.gov/snp/rs4880.

## Ethics Statement

The study was approved by the Institutional Review Board of the West China Second University Hospital, Sichuan University (2014-014 to PF). All of the participants provided their written informed consent in accordance with the Helsinki Declaration of ethical conduct in research.

## Author Contributions

PF conceived and designed the experiments, analyzed the data, and revised the paper. YS performed experiments and wrote the paper. HL and WH were responsible for patient screening. YG and YS collected samples and clinical data. QL and LG helped with the experiments. SL and HB helped with the experiments and revised the paper. All authors read and approved the final manuscript.

### Conflict of Interest Statement

The authors declare that the research was conducted in the absence of any commercial or financial relationships that could be construed as a potential conflict of interest.
